# Supplementary figures and images for: Ventilation-Based Strategy to Manage Intraoperative Aerosol Viral Transmission in the Era of SARS-CoV-2
Source: Life (Basel). 2024 Feb 28;14(3):313. doi: 10.3390/life14030313 (PMC10970813; doi:10.3390/life14030313)

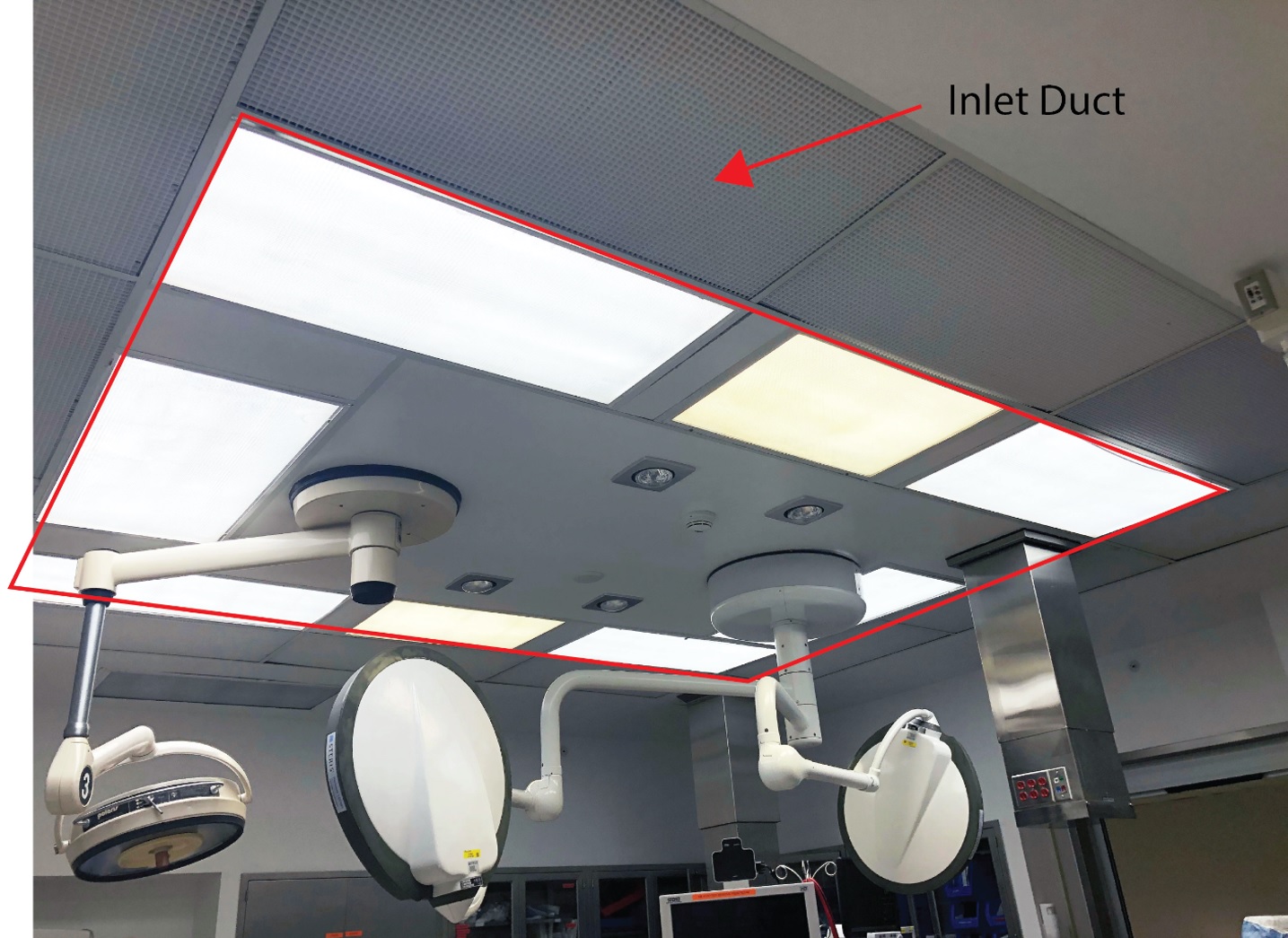

Supplement: Supplementary file 1 [file life-14-00313-s001.zip › Figure S1 Existing operating room inlet configuration adapted for configuration C2.jpg]
